# Insight and Inner Peace in Palliative Care Professionals after an Art Therapy Workshop Focused on Personal Self-Care: A Preliminary Experience

**DOI:** 10.1089/pmr.2020.0079

**Published:** 2021-02-08

**Authors:** María Arantzamendi, Paula Sapeta, Nadia Collette, Ana Baños Sesma, María Teresa Torres Pérez-Solero, Fernando Iribarren Echarri, Carlos Centeno

**Affiliations:** ^1^ Instituto Cultura y Sociedad (ICS), Universidad de Navarra, Grupo ATLANTES, Pamplona, España.; ^2^ IdisNA—Instituto de Investigación Sanitaria de Navarra, Pamplona, España.; ^3^ Escola Superior de Saúde Dr. Lopes Dias, Instituto Politécnico de Castelo Branco, Castelo Branco, Portugal.; ^4^ Unidad de Cuidados Paliativos, Hospital Santa Creu i Sant Pau, Barcelona, España.; ^5^ San Juan de Dios, Hospital Pamplona-Iruña, Pamplona, España.; ^6^ Museo Universidad de Navarra, Área Educativa, Pamplona, España.; ^7^ Departamento de Medicina Paliativa, Clínica Universidad de Navarra, Pamplona, España.

**Keywords:** art therapy, health care professional, health professionals, palliative care, qualitative research, self-care

## Abstract

***Background:*** Emotional exhaustion is a problem many palliative care (PC) professionals face during their activity. Art therapy is emotionally beneficial for palliative patients who experience suffering, but its impact on professionals' experience of suffering has not been researched.

***Objective:*** To examine the immediate reactions of professionals after an art therapy workshop focused on personal self-care, also considering previously used coping strategies.

***Design:*** A four-hour art therapy workshop was designed including a generic qualitative study of participants. Participants were PC professionals and their reactions were examined using an *ad hoc* questionnaire with open-ended questions. Descriptive analysis of quantitative variables and thematic analysis of open-ended questions were conducted.

***Results:*** Seventeen professionals participated voluntarily. They rated the workshop positively, using words such as “calm” and “relaxation” to express the effects of the workshop, which they considered therapeutic and a source of self-awareness. For some, it allowed them to release emotions; for others, it enabled introspection and opened up a more elaborated emotional response. They thought artistic expression would be useful for their colleagues, or even for their own personal development. In the workshop, professionals opened up and explained how they face intense moments on a day-to-day basis: how they approach the situation, or how they try to control their surroundings; how they disconnect/distance themselves; and how they consider circumstances as a learning process and source of self-nurturing. Participants described art therapy as calming, healing the most intense feelings, and feeding the soul.

***Conclusion:*** Professionals reacted immediately with enthusiasm to art therapy, positively assessing its effects. Some attributed effects are in line with daily strategies of connecting with one's inner self. Others are about promoting self-awareness and inner peace, while providing healing opportunities. Art therapy may play a role in self-care for the PC professional, and should be researched further.

Research Ethics Committee of the Universidad de Navarra approved the study (Number: 2019.167).

## Background

Palliative care (PC) professionals describe their work as both challenging and rewarding at the same time.^1^ They run the risk of emotional exhaustion and burnout due to the highly demanding nature of their work.^2,3^ Moreover, they need to cope with the challenges of the “self,”^4^ coping with their own emotions^5^ and existential queries,^6^ aspects that may affect not only their well-being but also the quality of their care.^7^

Art therapy is defined as a form of psychotherapy that uses mainly visual artistic media and that does not require any prior experience or artistic training on the part of the participant involved.^8–10^ Its general aim is to encourage a creative approach to emotional problems, to allow the person to change and grow personally in a safe and facilitating environment.^8–10^ It may help health care professionals to manage emotionally difficult situations, develop self-awareness, and promote self-care.^11,12^

The Navarrese Society for Palliative Care (PALIAN) organized an art therapy workshop for the region's professionals in collaboration with the Art Museum of the Universidad de Navarra and the ATLANTES Research Group.^13^ The art therapist had a master degree certified by the Spanish Federation of Professional Associations of Art Therapy. This country organization deals with the credentialing system for the association's registered art therapists to the highest qualified level of rigorous professional practice. The art therapist also had a bachelor degree in arts and a PhD in psychology.

A study was designed with the aim of examining the immediate reaction of professionals working with patients at end of life at an art therapy workshop focused on personal self-care. The study also took previously used coping strategies into consideration.

## Methods

This was a generic qualitative study^14,15^ designed to learn from the participants' experience, and their interpretation of their experience. The purpose was to generate new perspectives and hypotheses.^16^ The underlying hypothesis was how the process of reflection through art therapy may overlap or differ from prior coping strategies that PC professionals use.

### Setting

The Art Museum of the Universidad de Navarra is an innovative^17–23^ and significant learning environment^18^ where visitors can have meaningful experiences.^23–25^

### Subjects and recruitment

PC professionals voluntarily enrolled in the workshop, which was promoted through social media and by mail to the members of the Navarrese Society of Palliative Care.

#### Description of the workshop

A four-hour workshop was conducted by a certified art therapist starting off with a 30-minute reflective observation, contemplating selected art pieces, as perceptual stimuli to allow participants to connect with their inner self. This included a brief introduction on self-care, self-awareness, and basic concepts of art therapy: mind–body focus, secure relational space, appreciation of artistic capacity as a universal human aptitude versus artistic ability or talent, and the value of the creative process versus the aesthetic outcome, as a way to enable the person to have a different perspective and attitude. Next, different stages for undertaking artistic production followed, designed to promote insights into personal forms of coping, using a phenomenological transdisciplinary humanistic model.^26^ First, plastic expression was stimulated through body consciousness, that is, inviting participants to associate color, shape, and texture to the sensory experience. Then, an artwork was made from a clinical experience remembered as a therapeutic success and, finally, from an experience that the participant considered as a therapeutic failure. At each step, the participant summed up his/her creation in three or four words, which we termed “resonance words,” as a verbal extension of what the artwork suggested to him/her. After that, interpersonal interpretation was encouraged in small groups. Each participant received an additional resonance word from every other member of the group, in response to his/her artwork about therapeutic failure. To integrate artistic experience, a piece of individual creative writing in the form of a poem with one's own words and those received completed the creative process. Lastly, the artworks, the poem, and/or the feelings that emerged during the process were freely shared with the entire group.

### Data collection and analysis

Reactions on the workshop were gathered through an *ad hoc* questionnaire at the end (Supplementary Appendix SA1). To enhance participants' awareness of their coping responses and compare the emotional reactions arising in their art therapy process, this included issues around the workshop evaluation, the feelings and effects evoked, and the personal coping strategies in day-to-day practice. Finally, opinions about the potential of art therapy for professionals were elicited. Participants' sociodemographic data were collected.

After the workshop, the study was explained to participants and informed consent requested the study was approved by the Research Ethics Committee of the Universidad de Navarra Number: 2019.167.

The organizers compiled the questionnaires and two researchers analyzed the data through a descriptive analysis of quantitative variables and a thematic analysis of the open-ended questions. The two researchers coded the data based on their meaning, independently and inductively, without predefined categories. Initially each researcher coded the data. The two code lists were revised based on the data and, through consensus, a coding structure was agreed. In case of discrepancy, it was resolved by returning to the data. There were no major discrepancies. Repeated reading of the data helped to consider some possible overlap and differences between reflection through art therapy and professionals' prior coping strategies. The findings of the analysis are presented separately, facilitating the reader's assessment of it. This also prevented overinterpretation beyond the exploratory aim of the study.

## Results

All participants in the workshop agreed to participate. They worked in different types of PC services such as hospital PC support teams, inpatient PC units and PC home care teams, and mixed services (PC hospital and home care). The average age was 34.9 years (9.1 standard deviation). Regarding the experience in PC, nine participants had less than one year of experience, followed by between one to four years (*n*: 1) and five to nine years (*n*: 3). The more experienced group had between 15 and 20 years in PC (*n*: 3). They were doctors (*n*: 12), psychologists (*n*: 2), a nurse, a researcher (social worker), and an educator. Nine participants had no artistic background and the others mentioned music (*n*: 4) and painting (*n*: 4).

### Evaluation of the workshop

All participants said they would like to take part in future workshops and found the workshop useful. Participants liked most the quality of the trainer, her skills, and dynamic nature; the diversity of artistic materials and tools used; and most importantly, the atmosphere, which was described with words such as safe, personal space, freedom, calm, the chance to listen, trust, spontaneity, sincerity, and time to reflect. All of these aspects promoted the judgment-free expression of feeling, introspection, and reflection.

Among suggestions for improvement, the participants mentioned the limited time; the size of the groups; the fact that there was no break; and that it would be ideal to have several sessions.

### Identification of feelings and effects

**FIG. 1.**
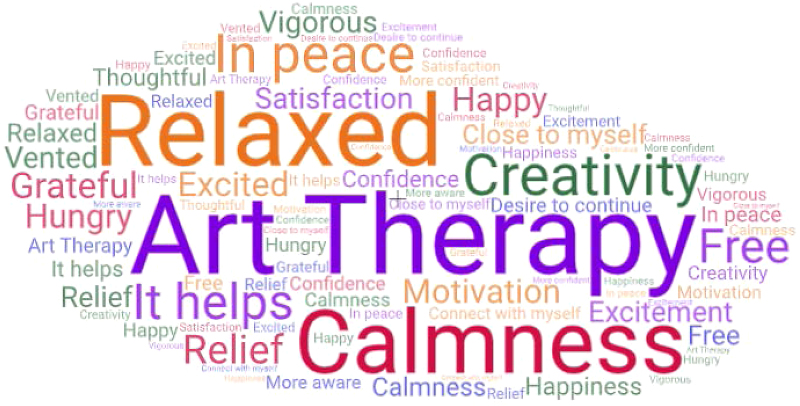
Word cloud: feelings aroused by the art therapy course.

The professionals described how they felt after doing the workshop using their own words (Fig. 1). The participants considered it useful and explained why. From the thematic analysis, we identified the following effects of art therapy in participants' accounts: (1) it allows expression of emotions that people have experienced, (2) it facilitates reflection and self-awareness, and (3) it has other therapeutic effects on the individual (Table 1). These echo coping strategies used by health care professionals.

**Table 1.** Effects of Art Therapy for the Palliative Care Professional Participating in a Self-Care Focused Workshop (Thematic Analysis of the Opinions of 17 Professionals)

Subject	Expressions
Allows expression of emotions felt	It is a way of expressing oneself
	(Allows) explanation where words are not enough
	(Works as a way of) illustrating emotions
	(Helps) articulate and share reality, sensations, and feelings
	Permits the expression of inner experiences that would normally remain hidden
	(Allows) the expression of feelings to provide value judgments and to come to terms with them
Allows for reflection and self-awareness	(Helps) in the quest for self-awareness
	Facilitates improvisations
	Enhances the self-perception of emotions experienced throughout one's life
	Permits time to give over to reflection
	Helps go outside one's comfort zone
	Allows one to find oneself
	Allows for the discovery of valuable things within oneself
Has other therapeutic effect on the person	Facilitates relaxation
	Helps to focus
	Artistic expression comes forth from the deepest part of oneself and this has a healing effect
	They are exercises in an inner process that changes from suffering to peace.

### Identification of coping strategies

Fourteen participants completed this section (three participants did not directly care for patients at that point).

The professionals' narratives show that—as part of the process—there may be at least one type of more reactive response, and another, more elaborated or premeditated one. Moreover, in their accounts, they use temporal markers (i.e., “on a day-to-day basis,” “in the long run,” “before and after”), from which we may infer that coping is not static in nature, but rather a dynamic process.

Based on professionals' comments, it seems that there are at least two types of visible trends in the strategies they employ: one, actions (without reflection) or disconnection; and the other, learning and personal growth (which implies connecting with the inner self, seeking balance, or taking part in cultural and creative activities).

- Some professionals tend to *“not stop to think (…) you just face up to the situation.” (P.7)*
- For others, disconnecting from clinical practice as a way of releasing emotions and feelings through physical exercise, or by performing housework, breathing. or reading.
- A good number of them feel that coping involves a learning process: being the frequent witness of another person's suffering gives a renewed meaning to life and provides greater value, or helps you to find meaning in small things. It is a gratifying experience that provides strength. Mention of the team, as a space for companionship and further learning, is made by many.
- Self-nurturing and growing are a denser more complex coping strategy seen in the study participants with more professional experience (i.e.: 13, 17, 20 years in PC). It includes connection with the inner self, silent introspection, solitude outside the primary setting, and giving oneself time to engage in personal reflection. Some feel the need to feed their spiritual dimension, through prayer or seeking energy from nature. There is also mention of creative or cultural activities: writing stories, a diary, and the pursuit of knowledge or creating new projects.

### The potential value of art therapy

Participants stated that they would tell colleagues, patients, or relatives about art therapy or thought it might be useful for their own personal development or for use in their clinical practice (Table 2).

**Table 2.** Expressions of Commitment to Change after the Art Therapy Course

Some verbatim comments from participants
*I intend to talk about art therapy with patients*
*I would like to be able to transmit the power of art to my colleagues*
*I would like to apply it to clinical practice: to give patients and relatives the opportunity to express their emotions through other media*
*I can use art as a tool for communication*
*We should consider the possibility of introducing art to express feelings; to reflect on what we do each day in our work.*
*I would like to seek more contact with beauty*
*I should focus on situations in which I feel comfortable and feed off them.*
*Tomorrow, I am going to feel like painting when I leave work.*
*I should take 5 minutes a day to think about how I feel.*
*I can reflect more about what happened at a certain time.*
*I can breathe whilst I contemplate a work of art.*
*I will make a short artistic expression of the day overall; we all have a bit of an artist inside.*
*I will try to remember the words (some of them) which came out in personal work.*

## Discussion

The professionals who took part felt that the art therapy workshop helped them to promote self-care. In their words, it facilitated access to their inner world and emotions. This effect may be related to the positive, secure, and nonjudgmental environment, and the innovative approach of using a museum and contemplating works of art, which activates certain neuronal circuits involved in homeostats and intensity of experience of beauty, may have enhanced this effect.^27^ Our approach could lead to a better acceptance of artistic proposals, designed with the integrative intention of restoring harmony between body and mind. This effect of channeling emotions has been reported previously,^11,12^ and is in line with strategies trying to deal with daily emotional challenges. Professionals use coping strategies in their daily work to deal with its emotional demands. If we come to understand these demands better, it will be easier to assess whether art therapy might be useful to promote strategies that professionals already use, or whether it may add new options.

One could argue that expression of emotion and promotion of reflection and self-awareness are types of “therapeutic effect” that go beyond the specific types mentioned by some participants (i.e.: changes from suffering to peace). This kind of activity would seem to offer a therapeutic effect *per se*, related to the quest for inner balance when relaxing or when developing a healing process.

The results of our study suggest that art therapy might offer different effects beyond burnout,^11,28,29^ by helping participants perceive how people establish different types of relational connections or disconnections. Our participants said that art therapy facilitated disconnection and emotional release, allowing them to know themselves better and understand their own feelings: using art channels emotions that at other times would remain hidden away. This also fits with the use of art therapy to help express and communicate experiences and feelings better.^9,10^

Our results suggest that art therapy also promotes more elaborated reflection and introspection that may help in the quest for self-awareness. As stated in the Self-Awareness-Based Model of Self-care,^30^ clinicians' self-awareness is the key to a whole-person approach to self-care. The model suggests that self-awareness can mitigate compassion fatigue and burnout, and enable exquisite empathy and healing connections. These latter aspects recall strategies mentioned by PC professionals, such as taking part in a “learning process” or in a deeper sense, “nurturing.”

Regarding limitations, it must also be acknowledged that the participants, having enrolled voluntarily, might have had a positive predisposition toward artistic expression. However, it is clear that at least for some professionals, art therapy might have a role in certain strategies, such as managing or expressing oneself, learning to disconnect, growing in self-awareness, or even gaining sustenance by connecting with one's inner self.

## Conclusions

Professionals reacted with enthusiasm to art therapy, assessing its effects positively. Some of the attributed effects are in line with daily strategies of connecting with one's inner self. Others are about promoting self-awareness and inner peace, which might be useful for the self-care of the professional, while providing for some healing opportunities. Art therapy may play a role in self-care for the PC professional, a possibility that warrants further research.

## Supplementary Material

Supplemental data

## Supplementary Material

Supplementary Appendix SA1

## Abbreviations Used

PALIAN: Navarrese Society for Palliative Care
PC: palliative care
