# Supplementary material for: Insight and Inner Peace in Palliative Care Professionals after an Art Therapy Workshop Focused on Personal Self-Care: A Preliminary Experience
Source: Palliat Med Rep. 2021 Feb 8;2(1):34–9. doi: 10.1089/pmr.2020.0079 (PMC8241372; doi:10.1089/pmr.2020.0079)
Supplement: Supplemental data [file Supp_AppendixS1.docx]

Supplementary Appendix SA1: Survey Questions

| **Daily practice**  Sometimes, in the daily practice of the professional who cares for advanced patients at the end of life there are intense situations that are difficult to handle because of what it arouses within us.  “We want to explore the management of feelings, emotions, thoughts, etc. that arise inside each one of us in these intense situations and that sometimes go away and other times last or stay ”.  How do you do it? How do you handle it? What helps you? |
| --- |
| **Sociodemographic data**  Gender:  Age:  Profession:  Experience in Palliative: years. |
| **Evaluation of the workshop** |
| Was this workshop useful for you?  Yes/No  If so: Explain it: |
| What have you appreciated the most about the workshop? |
| What did you like least about the workshop? |
| What words (minimum 3 words) would you use to define how you feel now, as a result of having done this workshop? |
| Do you have any kind of artistic background in your life journey?  Yes/No  If so: Which one? |
| Tomorrow in your working day, what novelty (however minimal) would you say you would include, as a result of having done this workshop? |
| Would you like to get involved in more workshops like this one in the future?  Yes/No  **THANK YOU VERY MUCH** |
